# Sexuelle Grenzverletzungen und sexualisierte Gewalt – Wo finden queere junge Menschen Informationen und Unterstützung?

**DOI:** 10.1007/s00103-026-04214-w

**Published:** 2026-03-09

**Authors:** Jasmin Stehr, Martin Wazlawik

**Affiliations:** Fakultät V – Diakonie, Gesundheit & Soziales, Hochschule Hannover, Blumhardtstr. 2, 30625 Hannover, Niedersachsen Deutschland

**Keywords:** Sexualisierte Gewalt, Queere junge Menschen, Angebotsnutzung, Peer-Unterstützung, Digitale Medien, Sexualized violence, Queer young people, Use of services, Peer support, Digital media

## Abstract

**Einleitung:**

Queere junge Menschen weisen ein erhöhtes Risiko für sexualisierte Gewalterfahrungen auf. In diesem Zusammenhang mangelt es an zielgruppenspezifischen Informations- und Beratungsangeboten. Zugleich verfügen queere junge Menschen aber auch über zentrale Ressourcen wie soziale Unterstützung und Online-Räume. Der Beitrag untersucht, welche Ansprechpersonen, Informationsquellen und psychosozialen Unterstützungsangebote queere junge Menschen bei dem Thema sexualisierte Gewalt aufsuchen. Ein besonderer Fokus liegt auf der Rolle digitaler Zugänge.

**Methoden:**

In einer Online-Befragung (06/2023–01/2024) wurden 146 queere junge Menschen im Alter von 16 bis 27 Jahren zu ihren Anlaufstellen und ihrer Angebotsnutzung befragt. Die Daten wurden deskriptiv ausgewertet. Zusätzlich fanden 5 Gruppendiskussionen (11/2022–12/2023) statt, die partizipativ und mit der dokumentarischen Methode qualitativ ausgewertet wurden.

**Ergebnisse:**

Von den 146 Befragten nutzen 50,3 % Aufklärungs- oder Beratungswebsites von Organisationen und 35,2 % Profile und Postings von Organisationen bzw. Expert*innen zur Informationssuche. Freund*innen sind für 65,8 % die zentralen Ansprechpersonen. Die Befragten verfügen über umfassende Kenntnisse psychosozialer Unterstützungsangebote. Das qualitative Datenmaterial zeigt die zentrale Bedeutung von Peer-Unterstützung und Online-Räumen, aber auch Hürden und Herausforderungen auf.

**Diskussion und Fazit:**

Die Ergebnisse weisen auf das Potenzial digitaler Informations- und Beratungsangebote zu sexualisierter Gewalt hin. Eine Integration peerbasierter Unterstützung in professionelle Angebote erscheint vielversprechend, bedarf jedoch einer ressourcenorientierten strukturellen Verankerung.

## Abstract (en)

**Introduction:**

Queer young people are at increased risk of experiencing sexualized violence. In this context, there is a lack of target-group-specific information and counseling services. At the same time, queer young people also have access to key resources such as social support and online spaces. This article examines which contact persons, information sources, and psychosocial support services queer young people seek out regarding the topic of sexualized violence. A particular focus is placed on the role of digital access.

**Methods:**

In an online survey (June 2023–January 2024), 146 queer young people aged 16 to 27 were asked about their points of contact and their use of services. The data were evaluated descriptively. In addition, five group discussions (November 2022–December 2023) took place, which were evaluated qualitatively using a participatory and documentary method.

**Results:**

Of the 146 respondents, 50.3% use educational or counseling websites provided by organizations, and 35.2% use profiles and posts by organizations or experts in order to search for information. Friends are the main point of contact for 65.8%. The respondents have extensive knowledge of psychosocial support services. The qualitative data highlight the central meaning of peer support and online spaces but reveal barriers and challenges as well.

**Discussion and conclusion:**

The results point to the potential of digital information and counseling services on sexualized violence. Integrating peer-based support in professional services appears promising, but requires a resource-oriented and structural anchoring.

## Einleitung

Die Abwesenheit von sexualisierter Gewalt als Voraussetzung für sexuelle Gesundheit hat in den letzten Jahrzehnten zunehmend Beachtung gefunden. Mit der Ausweitung der Definition sexueller Gesundheit der Weltgesundheitsorganisation (WHO) im Jahr 2002 rückte der Schutz vor sexualisierter Gewalt in den Fokus internationaler wissenschaftlicher wie politischer Diskurse [1, 2]. Die Definition betont, dass sexuelle Gesundheit die „Möglichkeit für lustvolle und sichere sexuelle Erfahrungen, frei von Unterdrückung, Diskriminierung und Gewalt“ [2], umfasst, wodurch das Recht auf sexuelle Selbstbestimmung und das Recht auf den Schutz vor sexualisierter Gewalt zu essenziellen Aspekten der sexuellen Gesundheitsförderung wurden.

Eine Bevölkerungsgruppe, die in dieser Hinsicht hohe Vulnerabilitätsmerkmale aufweist, sind queere Jugendliche und junge Erwachsene. Bisherige Forschungsarbeiten deuten darauf hin, dass junge Menschen aus dem LSBTIQA*-Spektrum einem erhöhten Risiko ausgesetzt sind, sexualisierte Gewalterfahrungen mit und ohne Körperkontakt zu erfahren [3–5]. Demgegenüber stehen strukturelle Versorgungslücken und Herausforderungen in der Ansprache und Erreichbarkeit dieser Zielgruppe. Vielerorts mangelt es an queerspezifischen Angeboten [6, 7] und während spezialisierte Fachberatungsstellen gegen sexualisierte Gewalt zwar eine zentrale Ressource in der Beratung, Intervention und Prävention sexualisierter Gewalt darstellen, zeigen sich auch hier bis in die jüngste Vergangenheit Bedarfe und Weiterentwicklungspotenziale hinsichtlich der Adressierung und Erreichbarkeit queerer Zielgruppen [8]. Darüber hinaus wird, basierend auf dem Minority Stress Model [9, 10], davon ausgegangen, dass queere (junge) Menschen im Vergleich zur Gesamtbevölkerung zusätzlichen Stressfaktoren ausgesetzt sind. Das Erleben und die Antizipation von negativen Erfahrungen durch Stigmatisierung und Diskriminierung können dabei nicht nur zu Gesundheitsbeeinträchtigungen, sondern auch zu stärkeren Vorbehalten gegenüber institutionellen Angeboten beitragen [11, 12].

Parallel dazu verfügen queere junge Menschen jedoch auch über zentrale Ressourcen. Insbesondere Freund*innen und queere Communitys werden häufig als wichtige Quelle der Unterstützung genannt, aber auch digitalen Medien wird eine tragende Rolle zugeschrieben [13–15]. Während junge Menschen digitale Medien zunehmend zur Informationssuche im Bereich Sexualität nutzen [16], werden sie für queere junge Menschen nicht nur als anonym zugängliche Informationsquelle, sondern auch als Ort des Austausches, der Vernetzung und Identitätserkundung als besonders relevant hervorgehoben [15].

Im vorliegenden Beitrag wird daher untersucht, welche Ansprechpersonen, Informationsquellen und psychosozialen Unterstützungsangebote queere junge Menschen bei dem Thema sexualisierte Gewalt hinzuziehen. Ein besonderer Fokus wird dabei auf digitale Zugänge gelegt. Die Datenbasis bilden eine Online-Befragung und Gruppendiskussionen, die in dem vom Bundesministerium für Bildung und Forschung (BMBF) geförderten Projekt QueerPar durchgeführt wurden. Nach einer kurzen Darstellung der Studie und der Methoden werden ausgewählte Ergebnisse der Online-Befragung und der Gruppendiskussionen vorgestellt und diskutiert.

## Methoden

### Studie.

Das BMBF-geförderte Projekt QueerPar wurde von einem Verbund der Hochschule Hannover (HsH) und der Deutschen Gesellschaft für Prävention und Intervention bei Kindesmisshandlung, -vernachlässigung und sexualisierter Gewalt (DGfPI) von 2022 bis 2024 durchgeführt. Das Forschungs- und Transferprojekt zielte auf die Qualifizierung und Sensibilisierung der Beratungs- und Unterstützungssysteme für queere junge Menschen im Kontext sexualisierter Gewalt ab. Während sich das Teilprojekt der DGfPI den Perspektiven spezialisierter Fachberatungsstellen widmete, wurden in dem Teilprojekt der HsH die Perspektiven queerer junger Menschen fokussiert [17]. Mit einem qualitativen und quantitativen Forschungsansatz wurden ihre Angebotsnutzung, Beratungserfahrungen und -bedarfe sowohl allgemein als auch spezifisch für den Kontext sexualisierte Gewalt untersucht. Dazu wurde zum einen eine teilstandardisierte Online-Befragung und zum anderen Gruppendiskussionen mit queeren jungen Menschen im Alter von 16 bis 27 Jahren durchgeführt.

### Online-Befragung.

Ziel der teilstandardisierten Online-Befragung war es, empirische Einblicke in die Angebotsnutzung und Perspektiven von queeren jungen Menschen auf spezialisierte Beratungs- und Informationsangebote im Kontext sexualisierter Gewalt zu gewinnen. Dazu wurde ein Fragebogen entwickelt und mithilfe des Tools LimeSurvey (LimeSurvey GmbH,  Hamburg, Deutschland) als Online-Fragebogen umgesetzt. Der Fragebogen umfasste 30 Items zur Nutzung digitaler Angebote, zu relevanten Informationsquellen und Ansprechpersonen bei den Themen Sexualität und sexualisierte Gewalt^1^ sowie zu Erfahrungen und Einschätzungen hinsichtlich spezialisierter Unterstützungsangebote. Abschließend wurden soziodemografische Angaben erfasst. Sofern möglich, orientierten sich die Items an bereits bestehenden Untersuchungen zur Mediennutzung [18] und Jugendsexualität [16], um eine Anschlussfähigkeit zu gewährleisten. Die weiteren Items wurden basierend auf Erkenntnissen aus der Literatur [9–11, 19, 20], Feldarbeit und der qualitativen Erhebung entwickelt. Aufgrund forschungsethischer Abwägungen zum sensiblen Themenfeld sexualisierte Gewalt erfolgte die Fragebogenentwicklung nicht partizipativ.

Es wurde ein Pretest durchgeführt, um die Passung und Verständlichkeit sowie die Nutzungsfreundlichkeit des Online-Fragebogens zu prüfen. Dazu wurde eine Gelegenheitsstichprobe von *N* = 6 jungen Menschen aus queeren Kontexten rekrutiert, von denen 4 Feedback gaben. Es wurden Anmerkungen zu Formatierungen, der Lesbarkeit durch Screenreader und zu einzelnen Antwortoptionen bezüglich digitaler Angebote und Informationsquellen gemacht. Zudem wurde auf das Fehlen eines Hilfsangebots und eine ungünstige Kategorisierung der Dorf- und Stadtgrößen hingewiesen. Basierend auf diesem Feedback wurde der Fragebogen entsprechend angepasst und ergänzt. Die Pretest-Daten wurden daher nicht in der Auswertung berücksichtigt. Die Datenerhebung erfolgte schließlich von Juni 2023 bis Januar 2024. Die Teilnehmenden wurden per E‑Mail und Aufrufe in sozialen Medien rekrutiert. Vorab wurde hierfür eine umfassende bundesweite Recherche queerer Jugendgruppen, -zentren und Vernetzungskontexte durchgeführt. Der Link zu der Befragung wurde an potenzielle Teilnehmende und Multiplikator*innen gesendet. Die Adressierten wurden eingeladen, teilzunehmen – sofern sie zu der Zielgruppe gehören – und/oder die Einladung zu streuen. Einschlusskriterien waren ein Alter zwischen 16 und 27 Jahren und die Selbstidentifikation als queer. Nach Datenbereinigung lag eine Stichprobe von insgesamt 146 Personen vor. Die deskriptive Auswertung erfolgte mit der Statistik-Software SPSS (IBM, Armonk, NY, USA).

### Gruppendiskussionen.

Um ein differenzierteres Verständnis für die Handlungspraxis queerer junger Menschen bei Unterstützungs- und Informationsbedarf und für ihre Wünsche an die Angebotsgestaltung im Kontext sexualisierter Gewalt zu gewinnen, wurden weiterhin 5 Gruppendiskussionen (11/2022–12/2023) durchgeführt. Einschlusskriterien waren ein Alter zwischen 16 und 27 Jahren und die Selbstidentifikation als queer. Es wurde angestrebt, möglichst „Realgruppen“ [21] zu erreichen, die sich auch im Alltag begegnen. Die Akquise erfolgte daher über queere (Jugend‑)Zentren und Projekte. Die Gruppen bestanden aus 2–8 Personen im Alter von 16 bis 27 Jahren. Bei den Gruppen 1 bis 4 handelte es sich um Realgruppen aus Jugendzentren. Die Gruppe 5 kannte sich größtenteils vorab nicht, teilte aber einen Vernetzungskontext. Der Leitfaden wurde in Anlehnung an das Gruppendiskussionsverfahren nach Bohnsack [21] entwickelt.

Die Teilnehmenden wurden schriftlich und mündlich über Projekt, Ziel, Ablauf und Inhalte der Gruppendiskussionen, Datenschutz, Freiwilligkeit und die jederzeitige Abbruchmöglichkeit der Teilnahme informiert, bevor sie schriftlich ihre informierte Einwilligung gaben. Den Teilnehmenden wurde vorab versichert, dass zu keinem Zeitpunkt eigene Gewalterfahrungen erfragt werden, und es wurde über mögliche Belastungen aufgeklärt. Sofern gewünscht, wurden gemeinsam nonverbale Signale vereinbart. Vor und nach der Gruppendiskussion wurde auf weiterführende Unterstützung durch lokale Fachberatungsstellen gegen sexualisierte Gewalt hingewiesen und eine entsprechende Liste ausgehändigt.

Nach einer Einstiegsgesprächsphase, die durch offene Stimuli angeregt wurde und den Teilnehmenden eigene Relevanzsetzungen zum Thema „Unterstützung suchen und finden“ ermöglichte, folgte eine stärker leitfadenorientierte Phase, die Erwartungen und Wünsche an die Angebotsgestaltung im Kontext sexualisierter Gewalt eruierte. Die Gruppendiskussionen dauerten zwischen 45 min und 2 Stunden und wurden anschließend transkribiert und pseudonymisiert.

Ausgewertet wurde das Datenmaterial zum einen partizipativ durch queere junge Menschen anhand des DEPICT-Modells [22] und zum anderen von Projektmitarbeitenden mittels der dokumentarischen Methode nach Bohnsack [21]. Die spezifische Gruppeninteraktionsform in den 2 dyadischen Diskussionen wurde in der dokumentarischen Auswertung berücksichtigt. Die Auswertungsergebnisse wurden in einem partizipativen Fachforum und durch die Entwicklung des Online-Angebots queerstellen.de zusammengeführt, in dem queere junge Menschen Wissen zu sexualisierter Gewalt und Beratung finden.

## Ergebnisse

Im Folgenden werden ausgewählte quantitative Ergebnisse der Online-Befragung vorgestellt und durch qualitative Eindrücke aus den Gruppendiskussionen ergänzt und vertieft. Zunächst werden Informationsquellen queerer junger Menschen und der Stellenwert digitaler Zugänge beleuchtet. Anschließend werden relevante Ansprechpersonen und die Rolle von Peer-Unterstützung fokussiert, bevor abschließend Ergebnisse zu den Kenntnissen, Zugängen und Nutzungserfahrungen queerer junger Menschen hinsichtlich psychosozialer Unterstützungsangebote im Kontext sexualisierter Gewalt berichtet werden.

### Stichprobe

Die Stichprobe wies einen Altersdurchschnitt von 21,2 Jahren (*SD* = 3,3) auf. Über die Hälfte der Befragten (56,9 %) wohnte in einer Großstadt mit mindestens 100.000 Einwohner*innen. Die meisten waren Student*innen (43,2 %), gefolgt von Schüler*innen (22,6 %) und Auszubildenden oder Berufstätigen (jeweils 11 %). Von den 146 Befragten identifizierten sich 35,6 % als cis-weiblich, 34,9 % als nichtbinär bzw. ähnlich und 15,8 % als trans*, 9 % als cis-männlich, 7 % wählten die Antwortoption „Ich weiß es (noch) nicht“ und 3 % „Ich kann oder möchte mich keiner der genannten Kategorien zuordnen“. Als sexuelle Orientierung gaben 25,3 % pansexuell, 23,3 % bisexuell und 21,9 % homosexuell an. Jeweils etwa 10 % identifizierten sich als asexuell oder gaben an, sich keiner der genannten Kategorien zuordnen zu können oder zu wollen. Nur 2 % wussten ihre sexuelle Orientierung (noch) nicht und 1,4 % gaben eine heterosexuelle Orientierung an.

### Informationsquellen bei dem Thema sexualisierte Gewalt

**Abb. 1:**
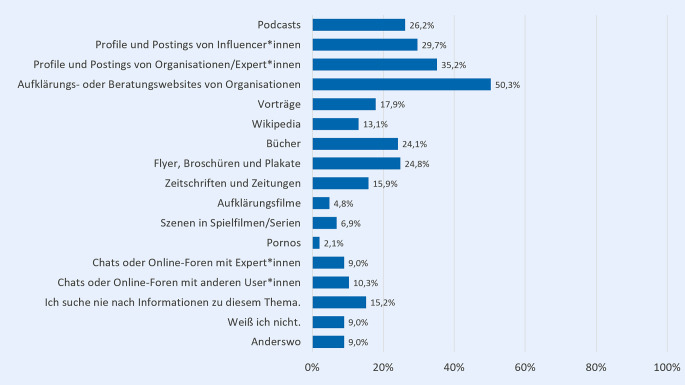
Informationsquellen bei dem Thema sexualisierte Gewalt (in %); *n* = 146; Mehrfachnennung möglich; Fragestellung: „Wo suchen Sie Informationen, wenn Sie etwas aus dem Bereich sexuelle Grenzverletzungen und sexualisierte Gewalt beschäftigt?“

Um sich in Bezug auf das Thema sexualisierte Gewalt zu informieren, nutzt jede*r zweite Befragte*r der Online-Befragung (50,3 %) Aufklärungs- oder Beratungswebsites von Organisationen (Abb. 1). Auch Profile und Postings von Organisationen bzw. Personen, die die Befragten als Expert*innen identifizieren, werden von über einem Drittel der Befragten (35,2 %) genannt. Auch Profile und Postings von Influencer*innen (29,7 %), Podcasts (26,2 %), Flyer, Broschüren und Plakate (24,8 %) sowie Bücher (24,1 %) sind unter den Befragten beliebte Informationsquellen. Etwas seltener werden dagegen Vorträge (17,9 %), Zeitschriften und Zeitungen (15,9 %) sowie Wikipedia (13,1 %) genannt. Ein Sechstel der Befragten (15,2 %) gibt derweil an, nie nach Informationen zu diesem Thema zu suchen.

Auch in dem qualitativen Datenmaterial zeigt sich, dass digitale Zugänge eine zentrale Rolle bei Informations- und Unterstützungsbedarf einnehmen. Digitale Medien werden dabei nicht nur für die Informationssuche als relevant benannt, sondern auch in ihrer vernetzenden und selbstbildenden Funktion thematisiert. Gleichzeitig berichten die Teilnehmenden jedoch auch von Erfahrungen sexualisierter Gewalt, die hier gemacht werden. So beschreibt eine teilnehmende Person (GD‑3, 407–416):

„Also vor allem auch beim Thema Sexualität. Ich bin damals, als ich mich so ’n bisschen selber gefunden habe, das war ungefähr mit vierzehn, auch in so WhatsApp-Gruppen gegangen, wo halt andere queere Menschen drin waren, weil es der einzige Ort war, wo ich wusste, dass ähm andere Menschen wie ich da sind. Und ähm vorher war die meiste sexuelle Belästigung nur von/von Typen und damals war/hab ich mich aber noch ähm als halt Frau identifiziert und dann kam das halt auch von/von Frauen irgendwie so nach’m Motto, hey, ich geh heute auf ein Date und willst du mir helfen bei dem, was ich anziehe und dann hab ich auf einmal äh fünf Unterwäsche-Bilder von dieser Frau bekommen und ich war so, oh, ich bin vierzehn und sie war definitiv über achtzehn.“

Das Erleben digitaler Medien zeigt sich in den Erzählungen der Teilnehmenden entsprechend ambivalent. Bieten sie einerseits ersehnte Austauschräume zur Erkundung der sexuellen oder geschlechtlichen Identität, bergen sie andererseits das Risiko für Erfahrungen sexualisierter Gewalt.

### Ansprechpersonen bei dem Thema sexualisierte Gewalt

**Abb. 2:**
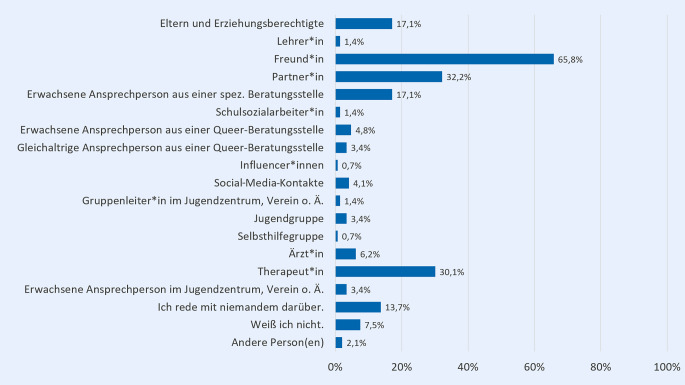
Ansprechpersonen bei dem Thema sexualisierte Gewalt (in %); *n* = 146; Mehrfachnennung möglich; Fragestellung: „Mit wem reden oder schreiben Sie, wenn Sie etwas aus dem Bereich sexuelle Grenzverletzungen und sexualisierte Gewalt beschäftigt?“

Auf die Frage, mit wem die Befragten reden oder schreiben, wenn sie etwas aus dem Bereich sexualisierte Gewalt beschäftigt, geben in der Online-Befragung mit Abstand die meisten die*den Freund*in (65,8 %) an (Abb. 2). Am zweithäufigsten genannt werden der*die Partner*in (32,2 %) und Therapeut*in (30,1 %). Im Vergleich dazu wendet sich nur etwa jede sechste Person (jeweils 17,1 %) an die eigenen Eltern und Erziehungsberechtigten oder an eine erwachsene Ansprechperson aus einer spezialisierten Beratungsstelle gegen sexualisierte Gewalt. 13,7 % geben an, mit niemanden über dieses Thema zu sprechen.

Die zentrale Bedeutung von Peers bei der Bearbeitung von herausfordernden Situationen spiegelt sich auch in den Gruppendiskussionen wider. Ob Peer-Unterstützung als präferierter Umgang oder eher als notgedrungener Ersatz beschrieben wird, steht dabei in einem engen Zusammenhang mit den zugrunde liegenden Verständnissen von professioneller Unterstützung und der Gewichtung von Erfahrungsexpertise: Während die einen Peer-Unterstützung bevorzugen, weil sie Erfahrungsexpertise einen hohen Stellenwert beimessen, sehen andere Peer-Unterstützung als Ersatzlösung, weil sie externe professionelle Unterstützung als unerreichbar erleben. In beiden Fällen herrscht jedoch ein Bewusstsein dafür vor, dass gegenseitige Peer-Unterstützung auch Herausforderungen bergen kann. So beschreibt eine teilnehmende Person (GD‑5, 40–57):

„Ähm weil, also zum Beispiel habe ich halt schon auch äh einfach mir eine Therapie gesucht und hatte auch eine Therapie, aber ähm hatte halt trotzdem nie so richtig das Gefühl, dass es da so wirklich verstanden oder mit bearbeitet wird, weil die Therapeut*innen halt, ja, das, also einfach aus den eigenen Lebensperspektiven nicht so richtig darauf spezialisiert sind, das nicht so richtig nachvollziehen können, ähm nicht so ne richtige Methodik haben irgendwie, das das zu bearbeiten. Und dann halt doch immer wieder vorwiegend so die eigene Community ähm (.) so, halt so ein Raum ist, wo man sich dann halt Unterstützung suchen kann. Was aber eben auch dazu führt, dass man sich schon gegenseitig auch immer wieder so ein Stück weit selber belastet …“

Angesichts des Risikos gegenseitiger Belastung zeigen sich in den Gruppendiskussionen das Suchen nach externer Unterstützung durch Fachkräfte sowie das aufmerksame und kritische Prüfen emotionaler Ressourcen bei sich selbst und anderen als Umgangsstrategien mit dieser Problematik.

### Kenntnisse, Zugänge und Nutzung von psychosozialen Unterstützungsangeboten im Kontext sexualisierter Gewalt

**Abb. 3:**
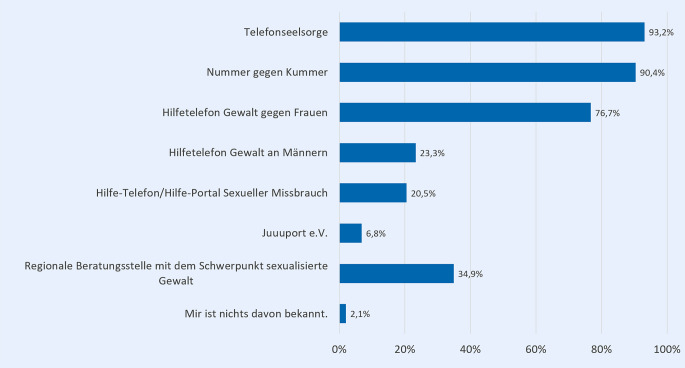
Kenntnisse über psychosoziale Unterstützungsangebote im Kontext sexualisierter Gewalt (in %); *n* = 146; Mehrfachnennung möglich; Fragestellung: „Von welchen dieser Angebote haben Sie schon gehört oder gelesen?“

Die Teilnehmenden der Online-Befragung wurden gebeten, aus einer Liste von psychosozialen Unterstützungsangeboten im Kontext sexualisierter Gewalt auszuwählen, welche ihnen davon bekannt sind. Der großen Mehrheit der Befragten ist die Telefonseelsorge (93,2 %) und die Nummer gegen Kummer (90,4 %) bekannt (Abb. 3). Auch das Hilfetelefon Gewalt gegen Frauen ist 3 Vierteln bekannt (76,7 %). Weniger bekannt sind dagegen Beratungsstellen mit dem Schwerpunkt sexualisierter Gewalt in der Region bzw. Nähe der Befragten (34,9 %), das Hilfetelefon Gewalt an Männern (23,3 %) und das Hilfe-Telefon bzw. Hilfe-Portal Sexueller Missbrauch (20,5 %). Den geringsten Bekanntheitsgrad hat das Online-Angebot Juuuport e. V. unter den Befragten (6,8 %). Lediglich 2,1 % der Befragten kennen keines der genannten Angebote.

**Abb. 4:**
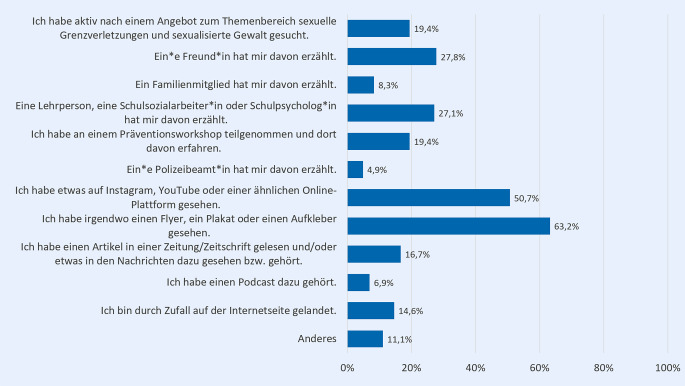
Zugänge zu psychosozialen Unterstützungsangeboten im Kontext sexualisierter Gewalt (in %); *n* = 144, nur Befragte, die mindestens ein Angebot kennen; Mehrfachnennung möglich; Fragestellung: „Wie haben Sie von diesen Angeboten erfahren?“

Diejenigen, die mindestens ein Angebot kennen (*n* = 144), wurden anschließend gefragt, wie sie von dem bzw. den Angebot(en) erfahren haben: 63,2 % geben an, irgendwo einen Flyer, ein Plakat oder einen Aufkleber gesehen zu haben, und 50,7 %, etwas auf Instagram, YouTube oder einer ähnlichen Plattform (Abb. 4). 27,8 % haben von den Angeboten durch ein*e Freund*in und 27,1 % durch eine Lehrperson, Schulsozialarbeiter*in oder Schulpsycholog*in erfahren.

**Abb. 5:**
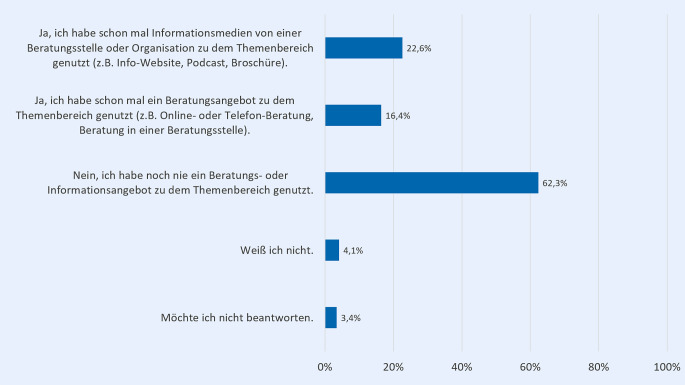
Bisherige Nutzungserfahrung spezialisierter Informations- oder Beratungsangebote gegen sexualisierte Gewalt (in %); *n* = 146; Mehrfachnennung möglich; Fragestellung: „Haben Sie schon mal ein Beratungs- oder Informationsangebot genutzt, das auf den Themenbereich sexuelle Grenzverletzungen und sexualisierte Gewalt spezialisiert ist?“

Blickt man auf die Nutzungserfahrungen der Befragten bezüglich spezialisierter Beratungs- oder Informationsangebote zum Thema sexualisierte Gewalt, zeigt sich, dass bereits 22,6 % Informationsmedien und 16,4 % schon mal ein Beratungsangebot von einer Beratungsstelle oder Organisation zu dem Themenbereich in Anspruch genommen haben (Abb. 5).

Ergänzend zu diesen quantitativen Einblicken zeigen die Gruppendiskussionen auf, dass der Weg von dem Wissen über psychosoziale Unterstützungsangebote bis zur Inanspruchnahme als voraussetzungs- und hürdenreich erlebt wird. So schildert eine teilnehmende Person (GD‑1, 71–82):

„… ja, auch, dass man irgendwie auch nicht weiß, dass es irgendwelche Stellen gibt, an die man sich wenden könnte, wenn man einfach äh nur so irgendwie dieses Gefühl hat, dass irgendwas falsch gelaufen ist, aber das nicht richtig einordnen kann ähm und dann irgendwie erst Jahre später erfährt, oh, da gibt’s sogar Stellen dafür, dass man sich da melden kann und das mit irgendwem besprechen kann. Ähm, (.) ja. Einfach, dass die Hilfsangebote nicht so (.) auf den ersten Blick sichtbar sind oder bekannt sind, ist halt irgendwie ein ziemlich großes Problem.“

Erfahrungen, die Gefühle des Unbehagens oder der Verunsicherung auslösen, müssen erst eingeordnet und als Phänomene benennbar werden, bevor die entsprechenden Unterstützungsangebote identifiziert und (auf-)gesucht werden können. Auch danach, so illustrieren die Aussagen der Teilnehmenden, werden Unterstützungssuchen weiterhin als hürdenreich erlebt. Lange Wartezeiten, veraltete Kontaktdaten und uneindeutige Informationslagen über die Queer-Sensibilität von Unterstützungsangeboten sind beispielhafte Hindernisse, die von den Teilnehmenden beschrieben werden. Entsprechend kommt der queerfreundlichen und übersichtlichen Außendarstellung von Angeboten in Abwägungsprozessen ein hoher Stellenwert zu. Pronomen-Angaben, queere Berater*innen und queere Symbole wie Flaggen sind beispielhafte Indizien, an denen die Teilnehmenden sich dabei orientieren.

## Diskussion

Die Ergebnisse der Online-Befragung und Gruppendiskussionen mit queeren jungen Menschen im Rahmen des QueerPar-Projekts geben Einblick in die Nutzung von Angeboten sowie die relevanten Ansprechpersonen und Informationsquellen zum Thema sexualisierte Gewalt. In den Ergebnissen der Online-Befragung wurde deutlich, dass queere junge Menschen informelle und formelle Ressourcen kennen und nutzen, um zu diesem Thema Unterstützung und Informationen zu finden. Die Gruppendiskussionen illustrieren dagegen die Hürden und Herausforderungen, mit denen sich queere junge Menschen bei Unterstützungssuchen dennoch konfrontiert sehen.

Der Großteil der befragten queeren jungen Menschen zieht Online-Quellen bei Informationsbedarf zum Thema sexualisierte Gewalt hinzu. Institutionelle Angebote wie Aufklärungs- und Beratungswebsites oder Profile bzw. Postings von Organisationen und Expert*innen sind dabei die beliebtesten Quellen. Vergleicht man diese Befunde mit den Erkenntnissen der Studie zu Jugendsexualität von 2019 der damaligen Bundeszentrale für gesundheitliche Aufklärung (BZgA), so deuten sich hier Parallelen an. Unter den 14- bis 25-Jährigen, die in diesem Rahmen befragt wurden, gehören Aufklärungs- und Beratungswebsites sowohl unter den heterosexuellen als auch unter den homo-, bisexuellen oder unentschlossen orientierten Jugendlichen und jungen Erwachsenen ebenfalls zu einer der relevantesten Online-Quellen der Sexualaufklärung [5].

Vergangene Studien haben bereits betont, dass digitale Medien eine zentrale Ressource queerer junger Menschen darstellen, diese sich aber durchaus ambivalent ausgestalten [14, 23]. Die Gruppendiskussionen bestätigen dies. Wie Timmermanns [14] zeigt, stellt das Internet „keinen diskriminierungsfreien Raum dar“, was auch für Erfahrungen sexualisierter Gewalt gilt. Die vorliegenden Ergebnisse unterstreichen somit die Relevanz von Online-Räumen und weisen auf die Notwendigkeit hin, digitale Zugänge zu spezialisierten Angeboten gegen sexualisierte Gewalt weiter auszubauen. Gleichzeitig verdeutlichen sie die Ambivalenz von Online-Räumen.

Bezüglich relevanter Ansprechpersonen bei dem Thema sexualisierte Gewalt zeigt sich in den Ergebnissen der Online-Befragung der Freundeskreis als bevorzugte Personengruppe, bei der queere junge Menschen bei Bedarf Unterstützung suchen. Dies steht im Einklang mit verschiedenen Studien zum Kommunikationsverhalten junger Menschen in Bezug auf erlebte sexualisierte Gewalt [24–28] sowie zu sozialen Ressourcen queerer (junger) Menschen im Allgemeinen [12, 23, 29]. Die Gruppendiskussionen machen jedoch deutlich, dass sich das Erleben von Peer-Unterstützung differenziert ausgestaltet und sowohl als bevorzugte als auch als Ersatzlösung diskutiert wird. Die Sorge, sich durch gegenseitige Unterstützung auch gegenseitig zu belasten, ist dabei in allen befragten Gruppen präsent. Dies lässt sich zu Studien in Bezug setzen, die zeigen, dass Disclosure-Prozesse zwischen Gleichaltrigen beidseitig als Herausforderung erlebt werden [30, 31]. Vor diesem Hintergrund erscheint eine strukturelle Integration von Peer-Unterstützung in institutionelle Angebote vielversprechend – es bleibt aber kritisch zu prüfen, wie beteiligte Peers vor Belastungen geschützt und ihre Ressourcen nachhaltig gewahrt werden können.

Die Ergebnisse der Online-Befragung zu den Angebotskenntnissen deuten auf einen hohen Wissensstand unter den befragten queeren jungen Menschen hin: Lediglich 2,1 % der Befragten kennen keines der aufgelisteten Unterstützungsangebote. Im Vergleich zu der 9. Welle der Jugendsexualität-Befragung [24] weist die vorliegende Studie damit eine Stichprobe auf, die mit einem Unterschied von 28 bis 42 Prozentpunkten deutlich stärker für Unterstützungsangebote sensibilisiert ist. Dieser Unterschied könnte jedoch auch auf einen Sampling-Bias zurückzuführen sein (s. Limitationen) und müsste in zukünftigen Studien überprüft werden. Umso aufschlussreicher sind jedoch die Erkenntnisse zu den Zugängen, über die die Befragten von den Angeboten erfahren haben. Am häufigsten erfuhren die Befragten von den Unterstützungsangeboten über zufällige Zugangswege wie Flyer, Plakate und Aufkleber oder Instagram, YouTube oder ähnliche Plattformen.

Ergänzend dazu zeigt das qualitative Datenmaterial auf, dass die Kenntnis über Angebote zwar eine Voraussetzung für deren Nutzung darstellt, die tatsächliche Inanspruchnahme jedoch durch Hürden und Hemmschwellen erschwert wird. In den Aussagen der Teilnehmenden zeigt sich, dass Unklarheit darüber, ob ein Angebot queerfreundlich ist oder dort Diskriminierungs- und Stigmatisierungserfahrungen zu erwarten sind, ein wesentliches Hemmnis bei der Angebotsnutzung darstellt. In dieser Hinsicht bestätigen die Ergebnisse das Minority Stress Model [9, 10]. Um Unterstützungssysteme im Kontext sexualisierter Gewalt für queere junge Menschen erreichbarer zu gestalten, erscheint es entsprechend unerlässlich, diese für queere Zielgruppen zu sensibilisieren und zu qualifizieren sowie gleichzeitig Hemmschwellen aufseiten der Adressat*innen durch niedrigschwellige Angebote zu senken.

### Limitationen

Die Sampling-Strategie für die Online-Befragung stellt eine Kombination aus einer gezielten Auswahl und einem Schneeballverfahren dar, sodass keinesfalls von einer repräsentativen Stichprobe ausgegangen werden kann. Durch die Rekrutierungsmethode ist es möglich, dass die Studie vor allem queere junge Menschen erreicht hat, die gegenüber institutionellen Angeboten besonders aufgeschlossen sind. Zudem mag das kommunizierte Ziel der Studie – das Beratungs- und Unterstützungsangebot im Kontext sexualisierter Gewalt für queere junge Menschen zu verbessern – vor allem junge Menschen angesprochen haben, die sich mit diesem Thema aus verschiedenen Gründen bereits auseinandergesetzt hatten. Die Stichprobe weist tendenziell einen überdurchschnittlich hohen Bildungsgrad auf. Die Ergebnisse der Jugendsexualität-Studie deuten darauf hin, dass hinsichtlich der sexualbezogenen Mediennutzung und des Kenntnisstandes von Unterstützungsangeboten Unterschiede je nach Bildungsgrad zu erwarten sind [16, 24]. Entsprechend sind zukünftige Arbeiten mit einem größeren, repräsentativen Sample zu empfehlen, um hier differenzierte Analysen zu ermöglichen.

KI-basierte Angebote waren zum Zeitpunkt der Fragebogenentwicklung (Mitte 2022 bis Anfang 2023) noch nicht in der Alltagsnutzung etabliert und wurden daher nicht berücksichtigt. Zukünftige Erhebungen sollten sie mit einbeziehen. Ebenso wurden aus forschungsethischen Gründen die Anliegen für die Informations- oder Unterstützungssuchen nicht abgefragt. Eine differenziertere Betrachtung der Nutzung oder Nichtnutzung sowie des Erlebens von Angeboten ist aufgrund der vorliegenden Daten daher nicht möglich.

## Fazit

Die vorliegende Studie bietet einen explorativen Einblick in die sozialen, medialen und institutionellen Ressourcen queerer junger Menschen hinsichtlich des Themas sexualisierter Gewalt. Die quantitativen Ergebnisse zeigen auf, dass queere junge Menschen zur Informationssuche insbesondere professionelle Online-Quellen nutzen. Die qualitativen Ergebnisse verdeutlichen dagegen erneut, dass die Ressource Internet durchaus ambivalent erlebt wird und hier auch Erfahrungen sexualisierter Gewalt gemacht werden. Während Freund*innen in den quantitativen Befunden die prävalentesten Ansprechpersonen darstellen, problematisieren die Befragten in den Gruppendiskussionen das Risiko, dass Peer-Unterstützung auch mit gegenseitiger Belastung einhergehen kann. Bezüglich der Kenntnis von psychosozialen Unterstützungsangeboten deuten die Ergebnisse der Online-Befragung auf eine informierte Population hin, die vor allem über indirekte Zugangswege erreicht wird. Die qualitativen Eindrücke machen die Hindernisse und Abwägungsprozesse deutlich, wenn eine Angebotsnutzung in Betracht gezogen wird.

Insgesamt unterstreichen die vorliegenden Befunde die Notwendigkeit, zielgruppenspezifische Informations- und Beratungsangebote im Kontext sexualisierter Gewalt weiter auszubauen. Die Berücksichtigung digitaler Zugänge, die Sensibilisierung und Qualifizierung von Fachkräften sowie der Abbau von Hemmschwellen durch niedrigschwellige Angebote und eine einladende Außendarstellung erscheinen dafür besonders relevant.

Trotz der methodischen Einschränkung, dass es sich bei der vorliegenden Online-Befragung um eine nichtrepräsentative Stichprobe handelt und die Daten lediglich deskriptiv ausgewertet wurden, bietet sie erste Erkenntnisse zu den Perspektiven queerer junger Menschen auf Informations- und Unterstützungsquellen im Kontext sexualisierter Gewalt. Die vorliegende Triangulation quantitativer und qualitativer Daten bietet damit differenzierte Einblicke und Anhaltspunkte für die Fachpraxis, um wirksam und nachhaltig zur sexuellen Gesundheitsförderung dieser Bevölkerungsgruppe beizutragen.

## Data Availability

Die während der vorliegenden Studie erzeugten und analysierten Datensätze sind aus forschungsethischen Gründen nicht öffentlich zugänglich.

## Footnotes

1 Um unterschiedlichen Verständnissen der Befragten Rechnung zu tragen, wurde in der Befragung das Begriffspaar „sexuelle Grenzverletzungen und sexualisierte Gewalt“ verwendet und zu Beginn des Fragebereichs darauf hingewiesen, dass sexualisierte Gewalt körperlich und nichtkörperlich erfolgen kann. Zugunsten der Lesbarkeit wird hier ausschließlich „sexualisierte Gewalt“ verwendet.
